# Heterogeneity in Biodistribution and Cytotoxicity of Silver Nanoparticles in Pulmonary Adenocarcinoma Human Cells

**DOI:** 10.3390/nano10010036

**Published:** 2019-12-21

**Authors:** My Kieu Ha, Kyung Hwun Chung, Tae Hyun Yoon

**Affiliations:** 1 Center for Next Generation Cytometry, Hanyang University, Seoul 04763, Korea; hakieumy@hanyang.ac.kr (M.K.H.); cyung6767@gmail.com (K.H.C.); 2 Department of Chemistry, College of Natural Sciences, Hanyang University, Seoul 04763, Korea; 3 Institute of Next Generation Material Design, Hanyang University, Seoul 04763, Korea

**Keywords:** single-cell, toxicity, flow cytometry, silver nanoparticles

## Abstract

Cellular association of nanoparticles (NPs) and their resultant cytotoxicity are heterogeneous in nature and can be influenced by the variances in NPs’ properties, cell types, and status. However, conventional in vitro assays typically consider the administered NP dose and the averaged cellular responses based on the assumption of a uniform distribution of monodisperse NPs in homogeneous cells, which might be insufficient to describe the complex nature of cell–NP interactions. Here, using flow cytometry, we report observations of the heterogeneity in the cellular association of silver nanoparticles (AgNPs) in A549 cells, which resulted in distinct dose-response relationships and cytotoxicity. Type I and Type II cells were moderately associated with AgNPs but as the cellular AgNP dose increased, Type I cells remained viable while Type II cells became less viable. Type III cells did not have high affinity with AgNPs but were, however, the least viable. Transmission electron microscopic images revealed that the biodistribution and the released Ag^+^ ions contributed to the distinct toxic effects of AgNPs in different populations. This single-cell dose-response analysis approach enabled the examination of how differently individual cells responded to different cellular NP doses and provided insights into nanotoxicity pathways at a single-cell level.

## 1. Introduction

In recent years, there have been tremendous efforts to examine the potential hazards of nanoparticles (NPs) on the biological system, since the NPs used in consumer products and biomedical applications have been reported to pose adverse effects on the environment and human health [1,2,3]. Conventional in vitro assays have been very useful in nanotoxicity studies as they are simple, fast and inexpensive, but they may be misleading because they assume a uniform distribution of monodisperse NPs in homogeneous cell populations and, therefore, only consider the administered dose of NPs and the cellular responses averaged over a large number of cells. However, the interaction between NPs and cells is a very nuanced process, complicated by the NPs’ colloidal behavior, such as agglomeration, sedimentation and diffusion [4,5], and cells’ physiological state, such as cell cycle, adhesion and migration [6,7]. As a result, the biodistribution and cytotoxicity of NPs vary greatly in individual cells [8,9], and the heterogeneity in the cellular uptake and toxic effects of NPs should be considered in order to gain a more detailed understanding of cell–NP interaction.

A variety of quantitative and semiquantitative approaches have been employed to address the cell-to-cell variance in NPs’ uptake and toxicity. Fluorescence microscopy is the method of choice for the cellular localization of NPs, but they suffer from low-throughput and limited spatial resolution (200–500 nm) [10]. Inductively coupled plasma mass spectrometry (ICP-MS) allows the quantification of cell-associated NPs. One of its new applications, laser ablation ICP-MS, offers additional insights on subcellular localization, but this image-based method suffers from low throughput and relatively low sensitivity. Single-particle ICP-MS utilizes the time-resolved mode to enable direct quantification of the number concentration, size distribution and agglomeration state of NPs, but it is only suitable for NPs larger than 20 nm in diameter and the cell concentration has to be carefully optimized to avoid false detection of doublets [11].

Flow cytometry (FCM) is a high-throughput technique that can measure light scattering and fluorescence in single cells. FCM has been used to detect cellular uptake of NPs via monitoring the changes in fluorescence intensities [12]. For NPs that do not have fluorescence but have high scattering efficiency instead, the detection of their cellular association using the side-scattered (SSC) intensity is more appropriate, as the SSC intensity is related to the inner granularity of cells [13,14]. Our recent studies have introduced a quantitative approach to estimate the cellular content of non-fluorescent NPs by calibrating the SSC intensity in FCM with parallel quantification by ICP-MS [15,16].

Among the variety of currently available NPs, silver nanoparticles (AgNPs) have been widely used for therapeutic interventions and medical diagnosis as drug carriers, nanoprobes, bio-imaging and labeling agents [17]. Extensive studies have investigated the involvement of AgNPs in adverse biological effects, such as reactive oxygen species (ROS) generation, mitochondrial injury, DNA damage, cell-cycle arrest and apoptosis induction [18,19,20,21]. Herein, we wanted to investigate the cellular association and toxicity of AgNPs in A549 (human pulmonary adenocarcinoma epithelial) cells. A549 cell line was selected because there have been publications reporting that NPs cause pulmonary diseases when they enter the human body by accumulating in lung tissues [22,23]. Using FCM, we monitored simultaneously the fluorescence intensity of propidium iodide (PI) for cells’ membrane integrity and the SSC intensity for cellular AgNP uptake in single-cell events. From this information, we were able to analyze how much each cell was responsive to a specific accumulated amount of AgNPs. We also applied transmission electron microscopy (TEM) to investigate the localization of AgNPs in cells. Our study has revealed the vast heterogeneity in the single-cell dose-response relationship and contributed to the understanding of cell-NP interactions.

## 2. Materials and Methods 

### 2.1. Silver Nanoparticles (AgNPs)

The AgNPs used in this study were coated with branched-polyethyleneimine (bPEI) with 40 nm and 80 nm nominal core diameters (denoted as Ag^40^ and Ag^80^ NPs, respectively), purchased from NanoComposix (San Diego, CA, USA). 

### 2.2. Hydrodynamic Size and Zeta Potential Characterization of AgNPs

Dispersions of AgNPs were prepared in ultrapure deionized (DI) water and Roswell-Park Memorial Institute medium (RPMI) (Gibco, Thermo Fischer Scientific, Waltham, MA, USA) supplemented with 10% fetal bovine serum (FBS) (Gibco, Waltham, MA, USA) and 1% penicillin-streptomycin (PS) (Gibco, Thermo Fischer Scientific, Waltham, MA, USA). The hydrodynamic sizes and zeta potentials were measured using a Malvern Zetasizer (Nano-ZS, Malvern Instruments Ltd., Worcestershire, UK) following manufacturer’ instructions. Measurements were performed in triplicate.

### 2.3. Cell Culture

The A549 cell line (ATCC CCL-185) was obtained from Korea Biological Resource Center and routinely cultivated in RPMI complete media in T-25 flasks (SPL Life Sciences, Pocheon, Korea) at 37 °C and 5% CO_2_ in an incubator (Panasonic Corp., Osaka, Japan)

### 2.4. Measurement of Cellular Dose and Response by Flow Cytometry

Cells were seeded on 60 × 15 mm dishes (Corning, Inc., Corning, NY, USA) and allowed to adhere overnight in the incubator. Next, the cells were treated with Ag^40^ and Ag^80^ NPs at 30 μg/mL for 24 h. The exposure conditions were chosen so that cells would show some toxic response from the association with NPs but still maintained adequate viability (Figure S1). In the Ag^+^ ion exposure, a stock solution of 5 mg/mL AgNO_3_ was prepared by dissolving AgNO_3_ powder (Sigma Aldrich, St. Louis, MO, USA) in deionized water. The stock solution was then diluted to 5 μg/mL in dispersions of Ag^40^ NPs and added into cell culture dishes. After exposure, the supernatant was aspirated and cells were washed with Dulbecco’s phosphate-buffered saline (DPBS) (Welgene, Inc., Gyeongsan, Korea) to remove excess AgNPs. Cells were then harvested with trypsin-ethylenediaminetetraacetic acid (EDTA) (Gibco, Thermo Fischer Scientific, Waltham, MA, USA) and centrifuged at 200× *g* for 3 min. The supernatant was discarded and the cell pellet was resuspended in 3 µM propidium iodide (PI) (Invitrogen, Thermo Fischer Scientific, Waltham, MA, USA) and incubated for 15 min at room temperature.

The prepared cells were analyzed on the Accuri C6 flow cytometer (BD Biosciences, Franklin Lakes, NJ, USA) equipped with a 488-nm laser for excitation. Light-scattered intensity was monitored on the forward-scattering (FSC) and side-scattering (SSC) channels, while PI fluorescence was monitored on the FL2 channel (BP 585/40). The threshold for removing debris was set at 10^6^ FSC-H intensity. Singlet gating was undertaken based on the FSC-A and FSC-H intensities. FlowJo^TM^ software (FlowJo, LLC., Ashland, OR, USA) was used for data gating and visualization.

### 2.5. Measurement of Cell Viability by MTT Assay

The cytotoxicity of Ag^40^, Ag^80^ NPs and Ag^+^ ions in A549 cells was examined by MTT (3-(4,5-dimethylthiazole-2-yl)-2,5-diphenyl tetrazolium bromide) assay. Cells were seeded in a 96-well plate and allowed to adhere overnight in the incubator at 37 °C and 5% CO_2_. Cells were then exposed to 30 μg/mL dispersions of Ag^40^ and Ag^80^ NPs, as well as 5 μg/mL Ag^+^ solutions. After 24 h, cells were treated with a solution of MTT (Sigma Aldrich, St. Louis, MO, USA) for 2 h. The MTT solution was then discarded and dimethylsulfoxide (Sigma Aldrich, St. Louis, MO, USA) was added to dissolve the insoluble formazan product. After 30 min, the supernatant was transferred to a new 96-well plate, and the absorbance was recorded on a microplate reader (GloMax^®^ Explorer, Promega, Madison, WI, USA) at 600 nm wavelength. Measurements were performed in triplicate for statistical analysis.

### 2.6. Sorting and Imaging of Different Cell Populations

Cells were seeded on culture dishes and allowed to adhere overnight in the incubator. Since the 3 populations were separated most clearly in the samples treated with Ag^40^ NPs, we decided to perform sorting and imaging on these samples. One sample was treated with 30 μg/mL Ag^40^ NPs, while another sample was treated with 30 μg/mL Ag^40^ NPs spiked with 5 μg/mL Ag^+^ ions for 24 h. After exposure, cells were washed with DPBS, harvested with 0.25% trypsin-EDTA and resuspended in DPBS containing PI and incubated for 15 min at room temperature. The samples were sorted on FACSAria III (BD Biosciences, Franklin Lakes, NJ, USA) based on their SSC and PI intensities. An unstained control and a stained negative control were also prepared and analyzed as references for gating. The sorted cells were further subjected to imaging analysis using TEM.

To prepare thin-sectioned specimens for TEM analysis, the sorted cells were fixed with 2.5% glutaraldehyde and 1% paraformaldehyde in DPBS for 3 h at 4 °C and then washed with DPBS. After that, the samples were post-fixed in 1% osmium tetroxide (Sigma Aldrich, St. Louis, MO, USA) for 2 h at room temperature and washed again with DPBS. Next, dehydration was performed by a graded ethanol series (50%, 60%, 70%, 80%, 90% and 100% ethanol) for 1 h each time. Cells were subsequently infiltrated by mixtures of ethanol and propylene oxide at 2:1, 1:1, 1:2 and 0:1 ratios for 1 h each, and then by mixtures of propylene oxide and epoxy resin (Structure Probe, Inc., West Chester, PA, USA) at 2:1, 1:1 and 1:2 ratios for 1 h each. Then, cells were embedded in epoxy resin and loaded into capsules to polymerize at 60 °C for 48 h. Thin-sectioning was performed using a Leica EM UC7 ultramicrotome (Leica Microsystems, Wetzlar, Germany) and collected on copper grids. Images were acquired using a JEM-1400 Flash TEM (JEOL Ltd., Tokyo, Japan) at 120 kV.

### 2.7. Statistical Analysis

The data are presented as mean ± standard error of the mean (SEM) calculated by OriginPro (version 2016 b9.3.2.303 Academic, OriginLab Corp., Northampton, MA, USA). Statistical significance was assessed using Student’s *t*-test. *p*-value < 0.05 was considered significant.

## 3. Results

### 3.1. Characterization of AgNPs

Physicochemical properties of the two types of Ag NPs used in this study, including nominal core size, hydrodynamic size, and zeta potential are presented in Table 1. The hydrodynamic sizes in DI water were similar to the nominal core sizes, but in RPMI media they were larger than the nominal core sizes. The standard deviation of the size distribution also increased in RPMI media. These results indicated that RPMI media, with the presence of serum proteins, induced agglomeration of Ag NPs. The hydrodynamic size of Ag^40^ NPs in RPMI media was larger than that of Ag^80^ NPs, suggesting that Ag^40^ NPs were more agglomerated than Ag^80^ NPs. The zeta potentials for the two types of NPs were both highly positive in DI water due to the positive charge of the bPEI-coating molecule but in RPMI media, they changed to slightly negative due to the negative charge of the protein corona adsorbed onto the surface of the NPs.

### 3.2. Single-Cell Dose-Response Analysis by Flow Cytometry

**Figure 1:**
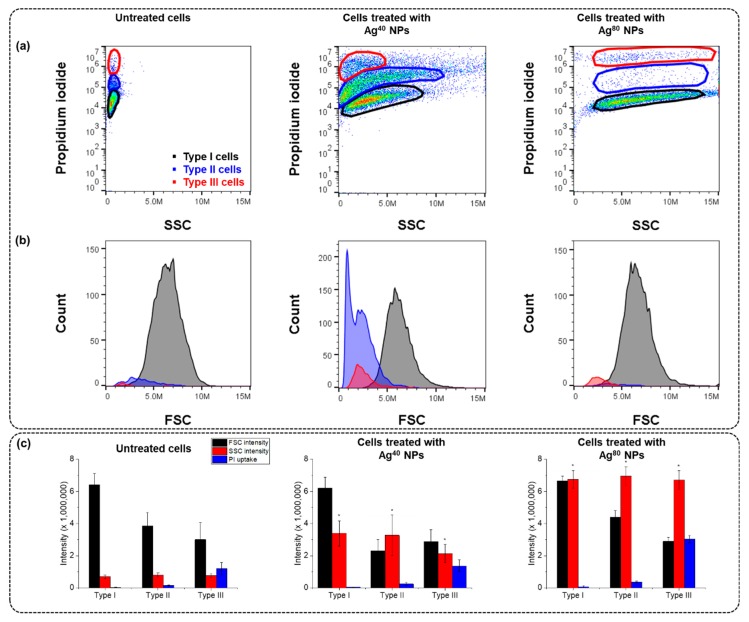
Cells treated with Ag^40^ nanoparticles (NPs) and Ag^80^ NPs in comparison with untreated cells. (**a**) Scatter plots of propidium iodide (PI) uptake against side-scattered (SSC) intensity; (**b**) histograms of forward-scattering (FSC) intensity; (**c**) Bar charts of averaged intensities of FSC, SSC and PI uptake in each population. Measurements were performed in 3 replicates. Data are presented as mean ± SEM. Student’s *t*-test was used to calculate statistical significance in comparison with control (*, *p* < 0.05).

As observed in Figure 1a, cells treated with Ag^40^ NPs separated into three distinct populations (denoted as Type I, Type II and Type III), while those treated with Ag^80^ NPs did not. These 3 populations were defined based on the arbitrary ranges of their PI uptake level. Type I was the healthy population which remained unaffected by the toxicity of Ag^40^ NPs as it exhibited low PI intensity (3 × 10^4^ a.u. on average as shown in Figure 1c) despite steady increments in the cellular AgNP dose. Type II was the mildly affected population as it showed an increase in the PI intensity as the cellular AgNP dose increased. However, the increase seemed to reach a plateau where the PI intensity fluctuated around 2.4 × 10^5^ a.u. after the SSC intensity exceeded 5 × 10^6^ a.u. Type III was the highly affected population as it displayed very high PI intensity (1.4 × 10^6^ a.u. on average) despite its low cellular AgNP dose (2.1 × 10^6^ averaged SSC intensity). Apart from the differences in SSC and PI intensities, the FSC intensity of each population also displayed distinct features (Figure 1b). FSC intensity is proportional to the size of cells, and changes in FSC intensity of treated cells in comparison to control cells reflect the swelling or shrinking of cells and may also provide us some hints about the toxicity of the treatment [16,24]. In the Ag^40^-treated sample, while the average FSC intensity of Type I cells was approximately 6 × 10^6^ a.u. which was similar to that of untreated cells, the average FSC intensities of Type II and Type III cells was only 2.3 × 10^6^ a.u. and 2.9 × 10^6^ a.u., respectively. The FSC histogram of Type II especially exhibited 2 overlapping peaks, one at around 1 × 10^6^ a.u. and one at roughly 2 × 10^6^ a.u. These observations indicate a negative correlation in the level of PI uptake and cell size, as Type II and Type III cells exhibited higher PI intensity and lower FSC intensity than Type I cells and the control sample. This shows that as the cells lost their membrane integrity, they shrank and/or generated apoptotic bodies, which corresponds with the morphological changes of Type II and Type III cells observed in confocal images in Figure S3. 

We applied similar gates to the untreated sample and the sample treated with Ag^80^ NPs to compare them with the sample treated with Ag^40^ NPs. There were significant differences in the cell count of each population between different samples. In the sample treated with Ag^40^ NPs, equal portions of cells were in Type I and Type II (i.e., 45% and 43%, respectively) and 11% were in Type III. In contrast, the majority of cells in the untreated sample (i.e., 87%) and the sample treated with Ag^80^ NPs (i.e., 78%) could be classified as Type I, while only small portions belonged to the other populations. Type II and Type III respectively accounted for 9% and 3% of the total number of cells in the untreated sample, 7% and 9% in the sample treated with Ag^80^ NPs.

In terms of SSC intensity, the average SSC intensity in the sample treated with Ag^80^ NPs reached 6.8 × 10^6^ a.u., higher than in the sample treated with Ag^40^ NPs, which was under 4 × 10^6^ a.u. on average for all three populations. This is understandable because the scattering intensity is proportional to the particle size [5,16], so cells treated with Ag^80^ NPs would have higher SSC intensity than those treated with Ag^40^ NPs. Our previous study also reported that the agglomeration state of NPs can affect the amount of cellular NPs [5]. As shown in Table 1, Ag^80^ NPs were less agglomerated than Ag^40^ NPs, which suggests that there were fewer NPs in an Ag^80^ agglomerate than in an Ag^40^ agglomerate. Therefore, when the agglomerates bound to the cell membrane or were engulfed in the endosomes, they involved more Ag^40^ NPs than Ag^80^ NPs.

**Figure 2:**
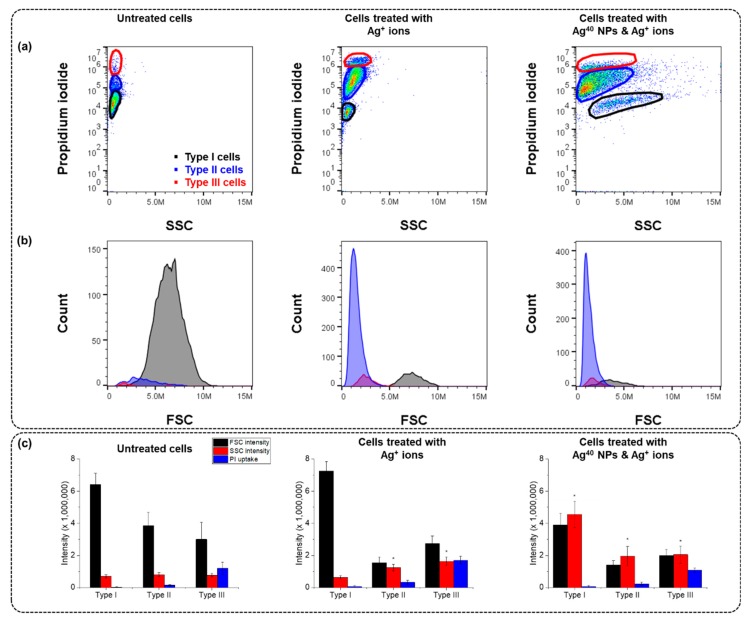
Cells treated with Ag^+^ ions and Ag^40^ NPs spiked with Ag^+^ ions in comparison with untreated cells. (**a**) Scatter plots of PI uptake against SSC intensity; (**b**) histograms of FSC intensity; (**c**) bar charts of averaged intensities of FSC, SSC and PI uptake in each population. Measurements were performed in 3 replicates. Data are presented as mean ± SEM. Student’s *t*-test was used to calculate statistical significance in comparison with control (*, *p* < 0.05).

We suspected that the cause of toxicity in Type II and Type III cells was contributed by Ag^+^ ions, as they have been reported to contribute to the toxicity mechanism of AgNPs in several studies [25,26,27]. Therefore, we analyzed cells exposed to an Ag^+^ ion solution or a dispersion of Ag^40^ NPs spiked with Ag^+^ (Figure 2). In the sample treated with Ag^+^ ions, the average SSC intensity was very low, around 10^6^ a.u., while in the sample treated with Ag^40^ NPs spiked with Ag^+^ ions, the average SSC intensity was higher, approximately 3 × 10^6^ a.u. This confirmed the increase in SSC intensity in the samples treated with AgNPs was indeed caused by the cellular association of AgNPs. Another interesting point is that the ratios of cells that could be classified as Type II and Type III in these 2 Ag^+^-treated samples were very high (78% and 83%, respectively), approximately 1.5-fold more than those in the Ag^40^-treated sample and 6-fold more than those in the untreated and Ag^80^-treated samples. We could conclude from these observations that the augmentation of Type II and Type III populations was contributed by the toxicity of Ag^+^ ions. 

**Figure 3:**
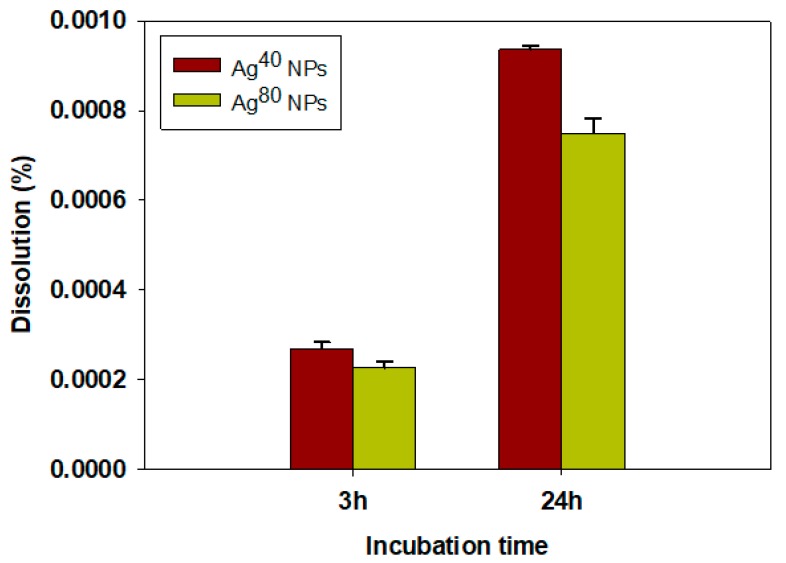
Percentage of dissolved Ag+ ions in dispersions of silver nanoparticles (AgNPs). Measurements were performed in 3 replicates. Data are presented as mean ± SEM.

We measured the dissolution of Ag^40^ and Ag^80^ NPs after 3 h and 24 h incubation in the cell culture media to estimate how much of the overall toxicity that the Ag^+^ ions accounted for and presented the results in Figure 3. There was obviously a time-dependency since dissolution after 24 h incubation time was nearly 5-fold higher than dissolution after 3 h. The percentage of released Ag^+^ ions for both types of AgNPs was very small, less than 0.001% of the administered dose which approximates 0.3 ng/mL, in all tested conditions. This explains why the ratios of Type II and Type III cells, which were suspected to be partly intoxicated by the Ag^+^ ions, were lower than that of Type I. The lower dissolution ratio of Ag^80^ NPs compared to Ag^40^ NPs also corresponded to the lower abundance of Type II and Type III cells in the Ag^80^-treated samples compared to the Ag^40^-treated ones. Because of such low dissolution of the AgNPs, the toxic effects observed in cells can be mainly attributed to the AgNPs, although the behavior of Type II and Type III populations suggested that the dissolved Ag^+^ ions still partly mediated the cytotoxicity of AgNPs.

To examine the interference that the free/unbound AgNPs in the samples might have on the detection of FSC, SSC, and PI, we analyzed the dispersions of Ag^40^ and Ag^80^ NPs without cells using FCM (Figure S2). The average FSC intensities of Ag^40^ and Ag^80^ NPs were below the cutoff threshold (10^6^ a.u.), while the average SSC and PI intensities were lower than those of most cells in the control sample. These observations confirm that the presence of free AgNPs in the samples did not interfere with the final data, as their signals were removed along with those of debris, and even if they had been included in the data, their SSC and PI intensities would not affect those of cell events.

### 3.3. Cellular Viability Analysis by MTT Assay

**Figure 4:**
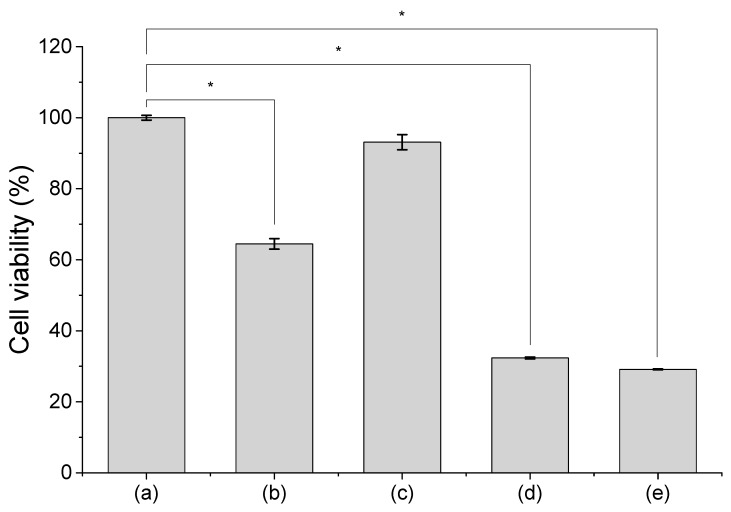
Viability of cells treated with Ag^40^, Ag^80^ NPs and Ag^+^ ions in comparison with untreated cells. (**a**) Untreated cells; (**b**) cells treated with Ag^40^ NPs; (**c**) cells treated with Ag^80^ NPs; (**d**) cells treated with Ag^40^ NPs spiked with Ag^+^ ions; (**e**) cells treated with Ag^+^ ions. Measurements were performed in 3 replicates. Data are presented as mean ± SEM. Student’s *t*-test was used to calculate statistical significance in comparison with control (*, *p* < 0.05).

To compare the results obtained from FCM to results from a more conventional method, we performed MTT assay to analyze the viability of cells treated with Ag^40^, Ag^80^ NPs, and Ag^+^ ions and presented the data in Figure 4. Cells treated with Ag^40^ NPs (Figure 4b) had a moderate viability of approximately 60%. This corresponded to the average PI intensity of the 3 distinct populations displayed in Figure 1. Because Type I, Type II and Type III cells respectively showed low, medium and high PI intensity, their average viability should be in a moderate range. In contrast, the viability of cells treated with Ag^80^ NPs (Figure 4c) remained high at 93%, which also corresponded to the high abundance Type I cells with low PI intensity. Cells treated with Ag^40^ NPs spiked with Ag^+^ ions (Figure 4d) and those treated with only Ag^+^ ions (Figure 4e) had viability of around 35%, which was a remarkable decline compared to control cells and also to cells treated with Ag^40^ NPs. This supports the findings in Figure 2, in which the majority of those samples could be classified as Type II and Type III with high PI intensity and only a fair number belonged to Type I with low PI intensity.

In general, results from FCM agree well with the observations from MTT assay. However, a disadvantage of the conventional MTT assay compared to the FCM technique was clearly demonstrated that it masked the heterogeneity in cellular AgNP dose and cellular response. Therefore, the single-cell capability of FCM provides a great advantage to the analysis of heterogeneity in cell–NP interactions.

### 3.4. Biodistribution Analysis of Different Populations by Transmission Electron Microscopy (TEM)

**Figure 5:**
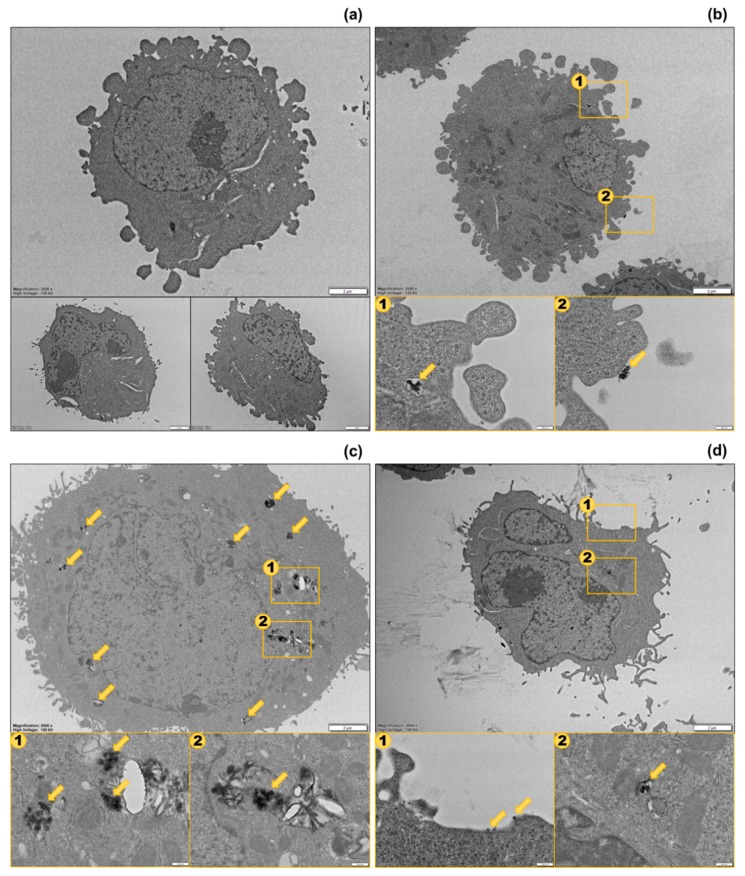
Thin-section images of sorted cells from transmission electron microscopy (TEM). (**a**) Untreated cells; (**b**) Type I cells; (**c**) Type II cells; (**d**) Type III cells. Scale bars in (**a**) are all 2 µm. Scale bars in (**b**–**d**) are 2 µm for large figures and 200 nm for small figures. The yellow arrows indicate the positions of cell-associated AgNPs.

To examine the distribution of the AgNPs in each cell population, we performed cell sorting based on SSC and PI intensities and observed the sorted cells using TEM. Since the 3 populations were separated most clearly in the samples treated with Ag^40^ NPs, we performed sorting and imaging on one sample treated with Ag^40^ NPs alone and another one treated with Ag^40^ spiked with Ag^+^ ions. Figure 5 displays TEM images of different populations. The AgNPs in both Type I and Type II cells were found in agglomerates, which agreed with their agglomeration status presented in Table 1. NPs in Type I cells (Figure 5b) could be observed on the membrane or in endosomes near the membrane, which suggests that the NPs were only membrane-bound or newly internalized. That is why they did not exert severe toxicity to cells. The membrane-bound AgNPs could have caused toxic effects by disrupting the cell membrane [28], but this possibility was low since our FCM data exhibited insignificant PI intensity in Type I cells. Meanwhile, the NPs in Type II cells (Figure 5c) were seen in endosomes in the cytoplasm, some were observed very close to the nucleus. The intracellular AgNPs and their released Ag^+^ ions were the cause of reduction in membrane integrity and viability in Type II cells. In Type III cells, NPs could hardly be seen. Figure 5d showed that some of them bound to the cell membrane as single particles while others were internalized as agglomerates. With so few cell-associated AgNPs but high PI uptake levels, Type III cells could be assumed to experience the toxic effects from the released Ag^+^ ions.

## 4. Discussion

Our FCM results in Figure 1 and Figure 2 display heterogeneity in A549 cells exposed to Ag^40^, Ag^80^ NPs and Ag^+^ ions that would otherwise become unnoticed in a conventional assay. Cells separated into distinct populations with unique features in the intensities of FSC (which is indicative cells’ size), SSC (cell’s granularity or AgNP uptake amounts in this case), and PI (membrane integrity loss), as well as morphology (as shown in their confocal images in Figure S3). Type I cells maintained their normal size, shape and membrane integrity although they were associated with many AgNPs. Type II cells experienced shrinkage and membrane integrity loss in proportion to the cell-associated AgNPs. Type III cells had elongated tails and lost their membrane integrity but did not have as much cellular AgNP association as the other two populations. The morphological changes in Type II and Type III cells corresponded with the decrease in their FSC intensity compared to Type I cells observed in Figure 1 and Figure 2. 

The presence of cells with intermediate and high PI uptake levels in Type II and Type III populations even in the untreated sample, although with very low abundance, indicates that cells are naturally heterogeneous. Such heterogeneity is inherent in cells, even for a cell line that is derived from a single clone with the same genetic background, and is driven by a number of factors such as cell cycle or cell migration [8,29]. The similarity in the ratios of cells in each population between the untreated sample and the Ag^80^-treated one indicates the low toxicity of Ag^80^ NPs, which kept the cellular heterogeneity nearly at the basal level. Meanwhile, the increase in the number of Type II and Type III cells (with intermediate and high PI uptake, respectively) in the Ag^40^-treated sample implies that Ag^40^ NPs were more toxic than Ag^80^ NPs. Cells with different status would interact and respond to Ag^40^ NPs differently, which intensified the distinction between the 3 populations. There are several reasons as to why different populations of the same cell line behaved differently when treated with AgNPs. One of the reasons we could think of is the differences in the biodistribution of the AgNPs. From the TEM images, we found that AgNPs mostly bound to the membrane in the Type I population. That explained why cells in this population had high SSC intensity but still maintained their viability. In the Type II population, many AgNPs were taken up inside the cells. This cellular population may suffer from the intracellular toxic effects from the internalized AgNPs and their dissolved Ag^+^ ions. In the Type III population, the AgNPs were hardly observed. We could assume that the presence of the Type III population resulted from the toxicity of Ag^+^ ions because they did not exhibit much cellular association with AgNPs but still experienced a great loss of membrane integrity. We also analyzed confocal images of each cell population, as in Figure S3, to investigate their potential changes in morphology. In Type I cells, the NPs were evenly distributed, cells still maintained their normal morphology as compared to control cells. Type III cells, on the other hand, had pointed and elongated tails but did not have much association with AgNPs. This suggests that the morphological change resulted from the toxic effects of the spiked Ag^+^ ions rather than of AgNPs. The elongated shape of Type III cells agrees with their FSC intensity (shown in Figure 1 and Figure 2). Because they were not as round and full as control cells, their FSC intensity decreased in accordance with the change in morphology. Meanwhile, large clumps of AgNPs could be seen in Type II cells. Their morphology was a fusion between Type I and Type III cells. They still maintained the overall round and full shape but started to have some pointed and elongated tails. As we already assumed the morphological effects of Ag^+^ ions, this observation implies the intracellular release of Ag^+^ ions from AgNPs and suggests that Type II cells suffered from the combined toxic effects of both particulate and ionic silver. According to De Matteis et al., Ag^+^ release was more efficient in the acidic intracellular environment than in culture media [27], thus the internalized AgNPs caused damage to Type II cells through the intracellular release of Ag^+^ ions.

Our FCM data also illustrate the size-dependent toxicity of AgNPs, as Ag^80^ NPs were found to be less toxic than Ag^40^ NPs, which agrees with results from conventional in vitro assays as in Figure 4. Based on the observation from TEM images, Ag^80^ NPs mostly bound to the cells’ membrane. That is partly why they could not exert their toxicity significantly. However, for the very few Ag^80^ NPs that were taken up inside of cells, their slow dissolution rate may have also contributed to their low toxicity. From a multi-omics study, Dekkers et al. reported that the modes of action of Ag and Zn NPs were mainly contributed by the dissolved metal ions, after observing that the molecular responses of A549 cells to these NPs were similar to their responses to Ag and Zn ions [30]. As displayed in Figure 3, Ag^80^ NPs indeed had less Ag^+^ ion release than Ag^40^ NPs and, therefore, induced lower toxicity. When the particle size goes smaller than 40 nm, it can be assumed that the toxicity will increase, as there have been several publications reporting the size-dependent toxicity of NPs [31,32]. Since the dissolution of NPs has also been reported to increase when the particle size decreases [33], we expect there will be more Type III cells, as they were the population mainly under the toxic effects of the dissolved Ag^+^ ions. These points were confirmed in the silver nanoclusters (AgNCs) with core sizes below 2 nm [34]. These AgNCs did exhibit higher cytotoxicity than AgNPs with 10–20 nm core sizes (i.e., LD_50_ of AgNCs at 24 h exposure in human neonatal foreskin fibroblast cells was 107 μg/mL compared to 340–430 μg/mL of 10–20 nm AgNPs in human periodontal fibroblast cells). The AgNCs also experienced dissolution in lysosomes, and therefore released Ag^+^ ions and induced the generation of reactive oxygen species (ROS). The elevated level of ROS can eventually result in oxidative stress, which is one of the important hallmarks of nanomaterial-induced cytotoxicity.

Our results demonstrate that the interaction between AgNPs and cells is vastly heterogeneous. Even in a cultured mammalian cell line that is normally considered homogeneous, certain populations are susceptible to the toxic effects of either AgNPs or Ag^+^ ions or a combination of both and other populations remain healthy despite high association with AgNPs. However, in these “healthy” populations, AgNPs may exhibit some effects that are non-toxic but still worth taking into consideration, such as the nanomaterial-induced endothelial leakiness (NanoEL). Titanium dioxide and gold NPs have been reported to bind directly to the VE-cadherin and disrupt cell–cell interactions, resulting in actin remodeling [35,36]. This effect does not depend on endocytosis of the NPs and precedes canonical hallmarks of NP-induced toxicity such as oxidative stress and apoptosis. This NanoEL effect adds to the heterogeneity in the cell-NP interactions another dimension that should be considered in future studies about the effects of NPs in biological systems. 

From the FCM data, we could develop a dose-response model for A549 cells exposed to AgNPs at a single-cell. Using bio-imaging as a complement technique, we could explain visually that dose–response relationship. Overall, we demonstrated the importance of investigating the cell-NP interaction at a single-cell level in order to understand the nanotoxicological pathways and mechanisms due to the observed heterogenous biodistribution of NPs and cellular response, which would normally be masked in a conventional in vitro assay.

## 5. Conclusions

In this study, we combined an estimation method for the cellular dose of AgNPs with the single-cell response metrics measured by FCM and demonstrated the single-cell dose-response relationship of AgNPs in A549 cells. Despite using one cell line, three populations with distinct toxicity levels were observed. Type I cells were insensitive to the association of AgNPs and remained healthy even at high cellular AgNP doses, while Type II cells were more vulnerable and became less viable than Type I cells, even under similar cellular dose conditions. Type III cells had the highest toxicity level among the three populations and were mostly intoxicated by the spiked Ag^+^ ions. From examining the TEM images of each population, we found that the biodistribution of AgNPs contributed to the separation of distinct cell types. With the cellular AgNP dose and the single-cell toxicity simultaneously measured by FCM, this study provides a basis for single-cell dose-response analysis and gives insights into the nanotoxicity mechanism. With the use of more quantitative techniques (e.g., mass cytometry, single-cell ICP-MS) for the estimation of cellular NP dose, this approach will help us understand better the heterogeneity of cell–NP interactions at a single-cell level.

## Figures and Tables

**Table 1:** Nominal core sizes, hydrodynamic sizes, and zeta potentials of Ag^40^ NPs and Ag^80^ NPs. Confidence intervals are the standard deviations of 3 replicate measurements.

Nanoparticle Type	Nominal Core Size ^1^ (nm)	Hydrodynamic Size ^2^ (nm)				Zeta Potential ^2^ (mV)	
		DI Water		RPMI Media			
		0 h	24 h	0 h	24 h	DI Water	RPMI Media
Ag^40^ NP	37 ± 4	48.8 ± 0.4	45.6 ± 0.3	153 ± 25	190 ± 22	74.7 ± 0.8	−8.5 ± 0.4
Ag^80^ NP	81 ± 9	84.4 ± 0.4	83.7 ± 0.9	108 ± 6	137 ± 16	32.5 ± 1.5	−11.7 ± 0.5

^1^ Confidence intervals indicate standard deviations of the size distribution. Nominal core sizes were referenced from manufacturer’s specifications. ^2^ Confidence intervals indicated standard deviations of 3 replicate measurements.

## Supplementary Materials

The following are available online at https://www.mdpi.com/2079-4991/10/1/36/s1: Figure S1: Dose-viability curves of A549 cells exposed to Ag^40^ and Ag^80^ NPs for 24 h measured by MTT assay; Figure S2: Dispersions of Ag^40^ and Ag^80^ NPs in comparison with untreated cells; Figure S3: Overlays of Hoechst fluorescence and brightfield images of sorted cells from confocal microscopy.
