# Supplementary material for: Heterogeneity in Biodistribution and Cytotoxicity of Silver Nanoparticles in Pulmonary Adenocarcinoma Human Cells
Source: Nanomaterials (Basel). 2019 Dec 21;10(1):36. doi: 10.3390/nano10010036 (PMC7022517; doi:10.3390/nano10010036)
Supplement: Supplementary file 1 [file nanomaterials-10-00036-s001.pdf]

nanomaterials

MDPI

Article

Heterogeneity in Biodistribution and Cytotoxicity of Silver Nanoparticles in Pulmonary Adenocarcinoma Human Cells

My Kieu Ha [1,2], Kyung Hwun Chung [1] and Tae Hyun Yoon [1,2,3,*]

1 Center for Next Generation Cytometry, Hanyang University, Seoul 04763, Korea; hakieumy@hanyang.ac.kr (M.K.H.); cyung6767@gmail.com (K.H.C.)
2 Department of Chemistry, College of Natural Sciences, Hanyang University, Seoul 04763, Korea
3 Institute of Next Generation Material Design, Hanyang University, Seoul 04763, Korea
* Correspondence: taeyoon@hanyang.ac.kr; Tel.: +82-2-2220-4593

1. Supplementary Methods

1.1. Measurement of Cell Viability by MTT Assay

The cytotoxicity of Ag^{40} and Ag^{80} NPs in A549 cells was examined using MTT viability assay. Cells were seeded in a 96-well plate and allowed to adhere overnight in an incubator at 37 °C and 5% CO_2. Cells were then exposed in triplicate to 0, 6.25, 12.5, 25, and 50 µg/mL dispersions of Ag^{40} and Ag^{80} NPs. After 24 h, cells were treated with a solution of 3-(4,5-dimethylthiazole-2-yl)-2,5-diphenyl tetrazolium bromide (MTT reagent, Sigma Aldrich, St. Louis, MO, USA) for 2 h. The MTT solution was then discarded and dimethylsulfoxide (Sigma Aldrich, St. Louis, MO, USA) was added to dissolve the insoluble formazan product. After 30 min, the supernatant was transferred to a new 96-well plate, and absorbance was recorded with a microplate reader (GloMax® Explorer, Promega, Madison, WI, USA) at 600 nm wavelength.

1.2. Dissolution Measurement

Dissolution of Ag^{40} and Ag^{80} NPs after being dispersed in DI water and RPMI media for 3 h and 24 h was measured by ICPMS. Ag^{40} and Ag^{80} NPs were diluted in DI water and RPMI media to make 0.5 mL dispersions, which were then transferred to centrifugal filters and centrifuge for 30 min at 13,500 rpm (12,225× *g*). The initial concentration of Ag NPs was 100 µg/mL. The filtered solutions were further diluted in 3% HNO_3 and used for ICPMS (NexION 300D, PerkinElmer Inc., Waltham, MA, USA) measurements to quantify the dissolved Ag^+ content. Three replicates were prepared for each Ag NP sample.

The Ag^+ ion standard samples (PerkinElmer Inc., Waltham, MA, USA) were used to generate a calibration curve for quantitative measurements. Based on the calibration curve and the measured ICPMS intensities, the dissolved Ag^+ content was determined, while the dissolution ratio was calculated as $[Ag^+]/[Ag\ NPs]_{initial} \times 100\%$.

1.3. Confocal Microscopic Image Acquisition

The samples treated with Ag^{40} NPs and with Ag^{40} NPs spiked with Ag^+ ions were sorted on FACSAria III (BD Biosciences, Thermo Fischer Scientific, Waltham, MA, USA) based on their SSC and PI intensities. The sorted cells were pelleted by centrifugation and resuspended in RPMI complete media, then allowed to adhere on coverslips pre-coated with poly-L-lysine (Sigma Aldrich, St. Louis, MO, USA). After that, cells were fixed with 4% paraformaldehyde (Wako Pure Chemical Industries, Ltd., Osaka, Japan) for 15 min and counterstained with Hoechst 33342 (Invitrogen, Thermo Fischer

Scientific, Waltham, MA, USA) for another 15 min. The coverslips were then mounted on microscope slides and observed under a confocal microscope (TCS SP5, Leica Microsystems, Wetzlar, Germany).

2. Supplementary Figures

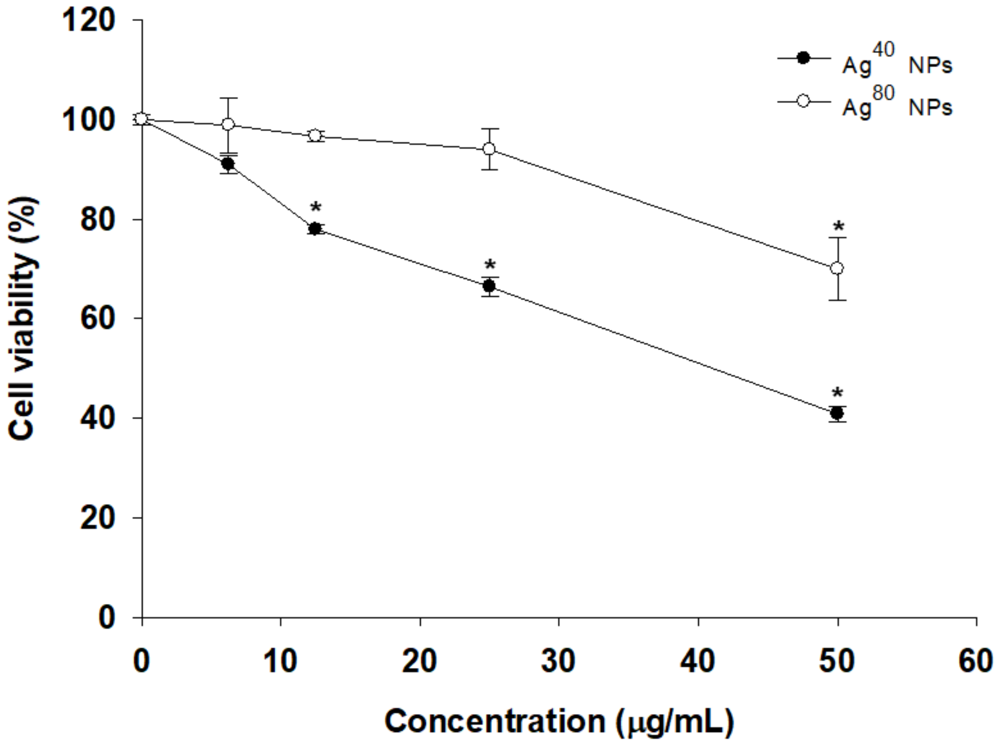

Figure S1. Dose-viability curves of A549 cells exposed to Ag^{40} and Ag^{80} NPs for 24 h measured by MTT assay. Measurements were performed in 3 replicates. Data are presented as mean ± SEM. Student's t-test was used to calculate statistical significance in comparison with control (*, $p < 0.05$). Ag^{80} NPs only had mild toxicity because cells exposed to them remained above 60% viable. Ag^{40} NPs were more toxic since the cell viability percentage decreased faster as the concentration increased and at 50 g/mL, it dropped to only 40%.

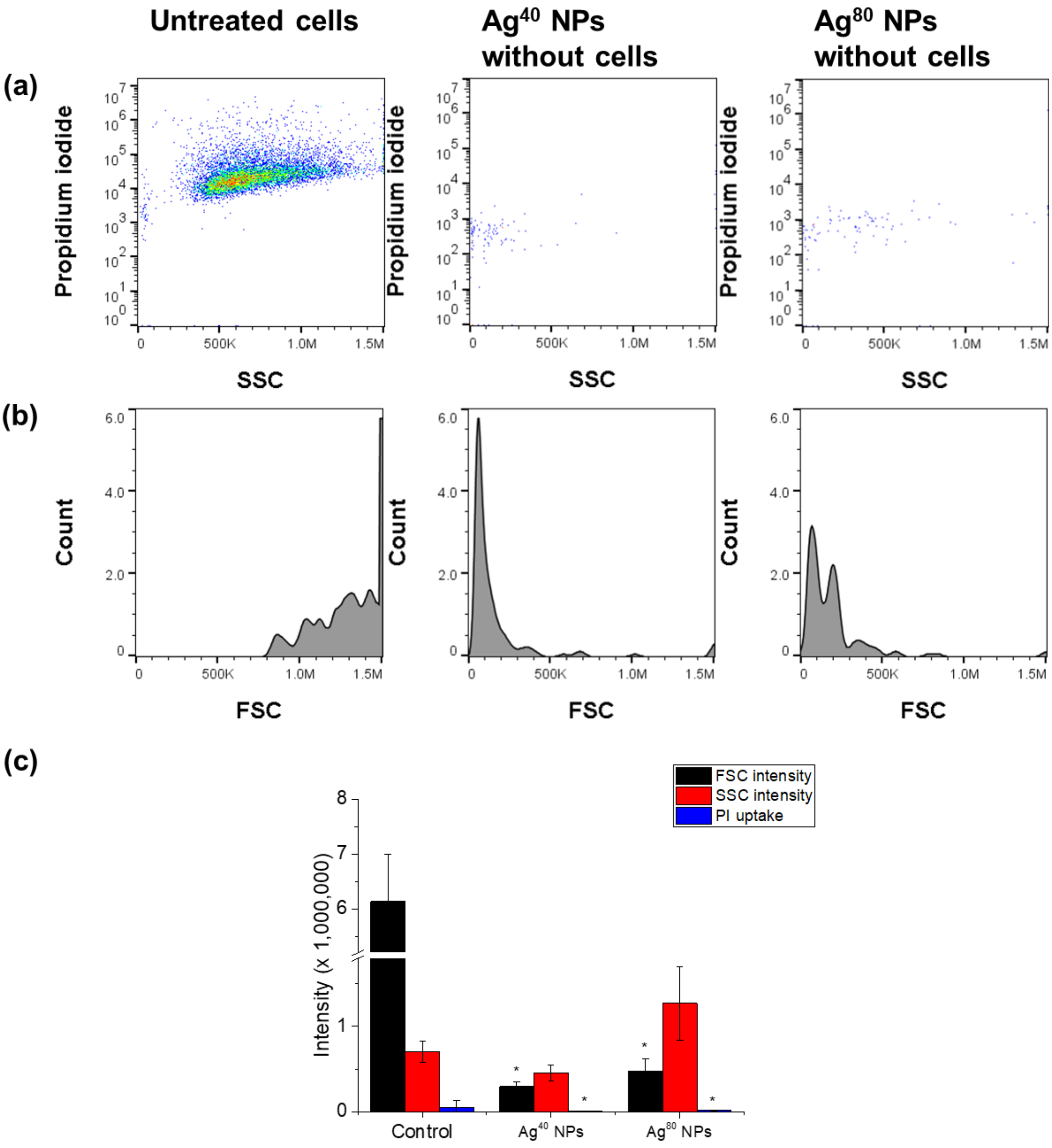

Figure S2. Dispersions of Ag^{40} and Ag^{80} NPs in comparison with untreated cells. (**a**) Scatter plots of PI uptake against SSC intensity; (**b**) Histograms of FSC intensity; (**c**) Bar charts of averaged intensities of FSC, SSC and PI uptake in each sample. Measurements were performed in 3 replicates. Data are presented as mean ± SEM. Student's t-test was used to calculate statistical significance in comparison with control (*, $p < 0.05$).

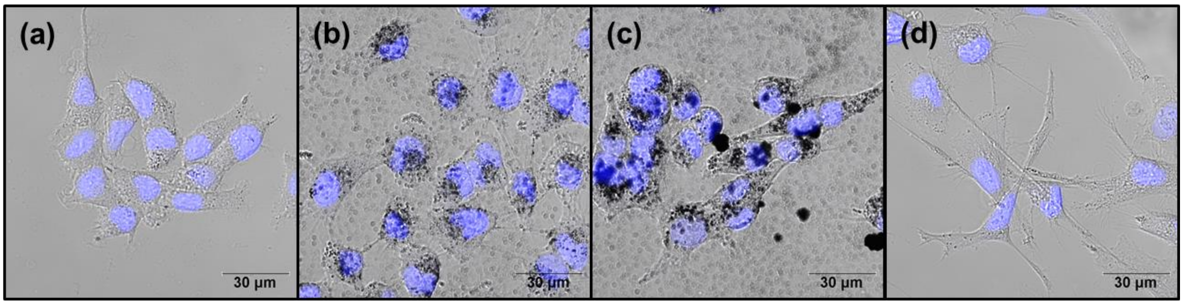

Figure S3. Overlays of Hoechst fluorescence and brightfield images of sorted cells from confocal microscopy. (**a**) Untreated cells. (**b**) In Type I cells, AgNPs were evenly distributed, cells still maintained their normal morphology as compared to control cells. (**c**) In Type II cells, there were large clumps of AgNPs, cells still maintained the overall round and full shape but started to have some pointy and elongated tails. (**d**) Type III cells were elongated in morphology and did not have much association with AgNPs.
